# iPSC-based merlin-deficient Schwann cell-like spheroids as an *in vitro* system for studying *NF2* pathogenesis

**DOI:** 10.1016/j.gendis.2025.101615

**Published:** 2025-03-27

**Authors:** Núria Catasús, Gemma Casals-Sendra, Miguel Torres-Martin, Inma Rosas, Bernd Kuebler, Helena Mazuelas, Emilio Amilibia, Begoña Aran, Anna Veiga, Ángel Raya, Bernat Gel, Ignacio Blanco, Eduard Serra, Meritxell Carrió, Elisabeth Castellanos

**Affiliations:** a Clinical Genomics Research Group, Germans Trias i Pujol Research Institute (IGTP), Can Ruti Campus, Badalona, Barcelona 08916, Spain; b PhD Program in Genetics, University of Barcelona, Barcelona 08007, Spain; c Genetics Department, Germans Trias i Pujol University Hospital (HUGTiP), Can Ruti Campus, Badalona, Barcelona 08916, Spain; d Medicine Regenerative Program, Institut d'Investigació Biomèdica de Bellvitge. IDIBELL, Hospital Duran i Reynals, Gran Via de L'Hospitalet, 199-203, L'Hospitalet de Llobregat, Barcelona 08908, Spain; e Hereditary Cancer Group, Germans Trias i Pujol Research Institute (IGTP), Can Ruti Campus, Badalona, Barcelona 08916, Spain; f Otorhinolaryngology Department, Germans Trias i Pujol University Hospital (HUGTiP), Can Ruti Campus, Badalona, Barcelona 08916, Spain

Neurofibromin 2 (NF2)-related schwannomatosis (NF2-SWN) is an autosomal-dominant tumor predisposition syndrome. NF2-SWN patients develop multiple benign tumors of the nervous system, such as schwannomas, particularly bilateral vestibular schwannomas, without current effective treatments.^1^ These tumors are caused by the bi-allelic inactivation of the *NF2* gene, which encodes for merlin protein, in a cell of the Schwann cell (SC) lineage.^2^ Changes in merlin result in the dysregulation of a wide variety of signaling cascades from the cell surface to the nucleus, such as the Hippo signaling pathway, by repressing YAP/TAZ nuclear translocation, and the FAK and PI3K/AKT/mTOR, Ras/Raf/MAPK, TP53, and Rac1-Pak1 pathways.^3^

Our current understanding of the molecular pathogenesis of NF2, as well as the development of new effective therapies, remains challenging due to the lack of non-perishable preclinical models that recapitulate the genetics and pathophysiology of human merlin-deficient SC and NF2 tumors. Induced pluripotent stem cells (iPSCs) constitute a suitable cellular model to solve this caveat thanks to their potential to differentiate into any cell type involved in NF2-SWN traits. With this aim, we generated iPSC lines with single or bi-allelic inactivation of *NF2* by combining the direct reprogramming of human primary vestibular schwannoma cells, with the use of CRISPR/Cas9 *NF2* gene editing (Fig. 1A). Genomic characterization of the generated *NF2*(+/−) and *NF2*(−/−) iPSC lines showed no differences with respect to the cells of origin, nor pathogenic off-target alterations of the edited lines, except for the induced NF2 variant. All clinical, genetic, and genomic information are summarized in Tables S1–4 and Figures S1 and 2. The *NF2* iPSC genotypes were further confirmed by evaluating merlin expression through Western blot analysis (Fig. 1B).

**Figure 1:**
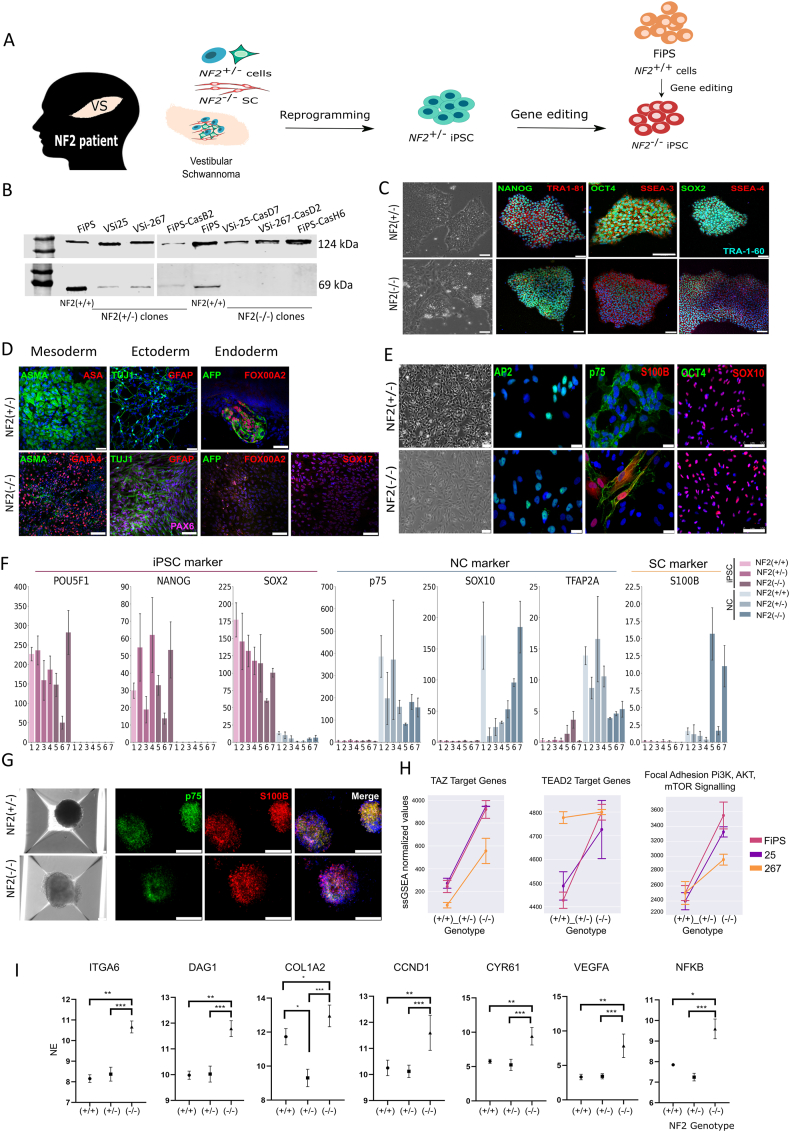
Merlin-deficient iPSCs can differentiate towards SC-like spheroids, showing dysregulation of multiple signaling pathways already described for *NF2*(−/−) SC, and altered in human schwannomas. (**A)** Schematic representation of the experimental procedure for obtaining merlin-null clones. **(B)** Merlin expression was analyzed by western blotting. The FiPS line generated from fibroblasts (*NF2*+/+) was used as a control cell line. **(C)** Morphology of iPSC colonies (left panel) and immunochemistry of pluripotency markers including NANOG, OCT4, and SOX2 (in green), as well as TRA-1-81, SSEA3 (in red), and TRA-1-60 (in cyan). Cell nuclei were stained with DAPI. Scale bar, 75 μM. **(D)** Immunochemistry was used to demonstrate the capacity of the lines to *in vitro* differentiate to the three primary germ layers of *NF2*(+/−) lines: mesoderm (ASMA in green and ASA in red), ectoderm (TUJ1 in green and GFAP in red), and endoderm (AFP in green and FOXA2 in red). For *NF2*(−/−), a directed differentiation was performed due to the inability of these lines to generate embryoid bodies, for mesoderm (ASMA in green and GATA4 in red), ectoderm (TUJ1 in green, GFAP in red, and PAX6 in pink), and endoderm (AFP in green, and FOXA2 and SOX17 in red). Scale bar, 75 μM. **(E)** NC morphology of *NF2*(+/−) and *NF2*(−/−) cell lines (left panel; scale bar, 75 μM) and immunocytochemistry of AP2 (green), p75 (green), and S100B (red) (scale bar, 25 μM); and Oct4 (green) and SOX10 (red) (scale bar, 100 μM. DAPI (blue) was used to stain cell nuclei. **(F)** Expression levels of the genes related to a pluripotent stage (POU5F, Nanog, and Sox2), an NC stage (p75, TFAP2A, and Sox10), and a SC marker (S100). Sample disposition: 1. *NF2*(+/+): FiPS, *NF2*(+/−); 2. VSi-25; 3. VSi-267; 4. FiPS-CasB2, *NF2*(−/−); 5. VSi-25-CasD7; 6. VSi-267-CasD2; 7. FiPS-CasH6 (see Table S2 for the information on iPSC lines). The bars express mean normalized expression (NE) ± standard deviation from three independent experiments. **(G)** Phase contrast images of *NF2*(+/−) and *NF2*(−/−) spheroids at day 7 of SC differentiation in 3D. No spheroids could be generated from the FiPS-CasB2 *NF2*(+/−) NC-derived cells (left panel; scale bar, 75 μM) and immunochemistry of p75 (green) and S100B (red) after 14 days of SC differentiation (scale bar, 250 μM). **(H)** The ssGSEA score in three individual pathways between *NF2*(+/+) and *NF2*(+/−) versus *NF2*(−/−) is shown. The error bars correspond to the standard error with the dot in the center as the mean. Samples from different genotypes were linked according to the source, one from the iPSC cell control line (FiPS), and two from vestibular schwannomas (25 and 267). **(I)** Gene expression analysis with VST values is shown for each gene. The bars express mean normalized expression ±standard deviation from three independent experiments. *t*-test was performed for each individual comparison among genotypes. Mean and standard deviation are shown. Significant comparisons are shown as ∗*p* < 0.05, ∗∗*p* < 0.01, and ∗∗∗*p* < 0.001.

*NF2*(+/−) and *NF2*(−/−) iPSC lines expressed cell surface proteins and transcription factors associated with pluripotency (Fig. 1C; Fig. S3), were positive for alkaline phosphatase staining, and showed karyotype stability after at least 20 passages (46, XY). However, whereas *NF2*(+/−) showed classical iPSC colony morphology, *NF2*(−/−) iPSCs presented less compact colony formation, eventually aggregating in the center of the colony, growing upwards and undergoing spontaneous differentiation (Fig. 1C). Furthermore, *NF2*(+/−) iPSCs showed the capacity to differentiate into the three primary germ layers *in vitro* through embryoid body formation, whereas *NF2*(−/−) lines required direct differentiation to acquire expression of the three germ layers (Fig. 1D; Fig. S3D and Table S5) and showed lower adherence capacity than their control *NF2*(+/−) counterparts (Fig. S3E). Nonetheless, despite the observed altered phenotype of *NF2*(−/−) iPSC lines, we were able to establish three *NF2* isogenic paired iPSC lines (*NF2*(+/−) and *NF2*(−/−)) with different genomic backgrounds and cells of origin.

Given that the cells that initiate schwannoma formation are *NF2*(−/−) cells of the SC lineage, we applied a differentiation protocol towards the neural crest (NC)-SC axis.^4^ After ten days of NC differentiation, cells achieved NC morphology, expressed NC markers, and repressed expression of pluripotency markers. However, *NF2*(−/−) NC cells showed population heterogeneity detected by flow cytometry and spontaneous expression of S100B, a marker of mature SCs (Fig. 1E, F; Fig. S4), indicating again altered behavior. Even so, they were able to maintain self-renewal capacity (>18 passages) and could be cultured after several freeze–thaw cycles. No differences in migration capacity were observed between the different genotypes (Fig. S4E), but reduced proliferation rates were observed in *NF2*(−/−) NC cells (Fig. S4F). Then, NC cells were differentiated to SC using standard 2D conditions. However, both *NF2*(+/−) and *NF2*(−/−) cells showed difficulties in maintaining adherence to culture dishes (Fig. S5). Therefore, we applied a 3D differentiation protocol and generated SC-like spheroids for up to 30 days.^4^ To evaluate the SC differentiation capacity of *NF2*(+/−) and *NF2*(−/−) cell lines in 3D, we studied the transcriptome at three time points (7, 14, and 30 days) of the differentiation process. *NF2*(+/+), *NF2*(+/−), and *NF2*(−/−) SC-like spheroids showed expression of typical SC lineage markers already at day 7 and up to 14 days of differentiation, such as CDH19, GAP43, EGR2, MPZ, PLP1, and S100B. However, at day 30 of differentiation, the expression of some SC markers decreased (SOX10 and PLP1) whereas some central nervous glial markers appeared (FABP7 and ASTN1), suggesting loss of SC commitment (Fig. S6). Expression of some of these markers was also corroborated by immunofluorescence analysis, showing homogenous expression throughout the spheroids (Fig. 1G). Finally, we confirmed the *NF2*(−/−) cell lines displayed a very similar *in vitro* NC-SC expression roadmap at 3D to that previously determined for *NF2*(+/+) differentiating SCs in 2D^4^ at day 7 and day 14 (Fig. S7). For all these reasons, we decided to focus the analysis at day 14 of differentiation (Fig. S8).

To better characterize *NF2*(−/−) SC-like spheroids and the effect of the absence of *NF2* on them, we performed differential expression analysis of the distinct genotypes at day 14 of SC differentiation. Only 125 genes were differentially expressed when comparing *NF2*(+/+) and *NF2*(+/−) genotypes, and most of them were related to cellular polarity and adhesion, to mTOR-pI3K-Akt pathway, or directly regulated by merlin (CHL1) (Fig. S9A, B). A higher number of differentially expressed genes were found when comparing the *NF2*(−/−) to *NF2*(+/−) or *NF2*(+/+) (2874 and 3447, respectively) (Fig. S9C, E), indicating that the complete *NF2* inactivation is the driver of the major expression changes in these cells. To determine whether this cellular model recapitulated the described pathophysiology of *NF2*-deficient SCs, we performed functional enrichment analyses, which showed that mTORC1, NFKβ, p53, Hedgehog, and IL6-JAK-STAT3 signaling pathways were significantly enriched in *NF2*(−/−) SC-like-spheroids when compared with *NF2*(+/−) or *NF2*(+/+) SC-like spheroids (Fig. S9D, F), as previously described.^3^

Sample gene set enrichment analysis (ssGSEA) identified, as expected on merlin deficient cells, that TAZ and TEAD2 target genes were up-regulated in *NF2*(−/−) SC-like spheroids, as did genes regulated by FAK and PI3K/AKT/mTOR pathways (Fig. 1H).^3^ We were also able to confirm that some of the major YAP and PI3K/AKT/mTOR pathway target genes, known to be altered in both *NF2*(−/−) primary SCs and schwannomas, were significantly up-regulated in *NF2* deficient SC-like spheroids. Moreover, alterations were found in well-known merlin-related cytoskeletal organization markers, such as Itga6, Dag1, and Col1a2^5^ (Fig. 1I; Fig. S10) which could compromise their ability to differentiate towards an SC identity in 2D culture conditions. Similarly, GSEA analysis revealed that other relevant merlin targets previously found to be altered in schwannomas were also up-regulated in the *NF2*(−/−) spheroids (Fig. S8, 10).

Altogether, these findings showed that the alterations identified in *NF2*(−/−) iPSC-derived SC-like spheroids could be attributed to the lack of merlin. Moreover, these results highlight a strong correlation between the previously described altered signaling pathways and gene expression profiles of merlin-deficient SCs and the ones observed in *NF2*(−/−) iPSC-derived SC-like spheroids, indicating that these cells, with single or bi-allelic inactivation of *NF2*, constitute a genuine *in vitro* human system for the study of the *NF2* role in SC, and potentially in any cell type associated with NF2-SWN pathogenesis.

## CRediT authorship contribution statement

**Núria Catasús:** Writing – original draft, Validation, Methodology, Investigation, Formal analysis, Data curation. **Gemma Casals-Sendra:** Methodology, Investigation, Formal analysis. **Miguel Torres-Martin:** Software, Formal analysis. **Inma Rosas:** Resources, Project administration. **Bernd Kuebler:** Methodology. **Helena Mazuelas:** Methodology. **Emilio Amilibia:** Resources. **Begoña Aran:** Methodology. **Anna Veiga:** Resources. **Ángel Raya:** Conceptualization. **Bernat Gel:** Visualization, Software. **Ignacio Blanco:** Resources, Funding acquisition. **Eduard Serra:** Writing – review & editing, Resources, Funding acquisition, Conceptualization. **Meritxell Carrió:** Writing – review & editing, Supervision, Methodology, Conceptualization. **Elisabeth Castellanos:** Writing – review & editing, Writing – original draft, Validation, Supervision, Resources, Project administration, Investigation, Funding acquisition, Conceptualization.

## Ethics declaration

This study was approved by the IGTP Human Research Ethics Committee (CEIC) (approval number: PI-17-250). Written informed consents were obtained from all participants to donate their samples to generate the iPSC used in the manuscript.

## Funding

This study has been funded by Chromo22; the *ISCIII* (No. PI20/00215, PI23/00619) (Co-funded by the European Regional Development Fund “A way to make Europe”), and AC22/00033, partner of the EJPRD. The EJPRD initiative has received funding from the European Union's Horizon 2020 research and innovation program under grant agreement No. 825575; funded also by *Fundació La Marató de TV3* (No. 126/C/2020), the Children's Tumor Foundation (No. CTF-2019-05-005, CTF-2022-05-005), Fundación Proyecto Neurofibromatosis, and the Government of Catalonia (No. SGR-Cat 2021-00967).

## Conflict of interests

The authors have no competing interests to declare.
