# Supplementary material for: iPSC-based merlin-deficient Schwann cell-like spheroids as an in vitro system for studying NF2 pathogenesis
Source: Genes Dis. 2025 Mar 27;12(6):101615. doi: 10.1016/j.gendis.2025.101615 (PMC12311431; doi:10.1016/j.gendis.2025.101615)
Supplement: Multimedia component 1 [file mmc1.docx]

# **MATERIALS AND METHODS**

All procedures performed were in accordance with the ethical standards of the IGTP Human Research Ethics Committee (CEIC) (approval number: PI-17-250), which approved this study, and with the 1964 Helsinki declaration and its later amendments. Written informed consents were obtained from all participants to donate their samples to generate the iPSC used in the manuscript. The “*Comisión de Garantías para la Donación y Utilización de Células y Tejidos Humanos, ISCIII*”) authorized the Project.

**Patients and Vestibular Schwannoma samples:** tumor samples were obtained from NF2-related SWN (NF2-SWN) patients clinically managed at the Spanish Reference Centre (CSUR) for phacomatoses HUGTiP-ICO-IGTP, diagnosed according to standard diagnostic criteria (1). Written informed consent was obtained for iPSC generation and genomic analysis.

**Tumor collection and cryopreservation:** tumor samples were placed in DMEM medium (Gibco) with 10% FBS, 1x GlutaMAX (Gibco) and 1x normocin antibiotic cocktail (InvivoGene) after surgery resection and cryopreserved in 10% DMSO (Sigma) + 90% FBS until processed.

**Tumor processing:** VS were digested with 160U/mL Collagenase type 1 and 0.8U/mL Neutral protease (Worthington, Lakewood, NJ) for 16 h at 37^o^C. Dissociated cells were seeded on 0.1 mg/mL Poly-L-lysine (Sigma) and 4 µg/mL laminin (Gibco)-coated dishes in Schwann cell medium (SCM) and maintained at 37^o^C and 10% CO_2_ atmosphere. SCM consists of: DMEM (Gibco) with 10% FBS, 500 U/mL penicillin/500mg/mL streptomycin (Gibco), 1x GlutaMAX (Gibco), 0.5mM 3-iso-butyl-1-methilxantine (Sigma), 2.5 µg/mL insulin (Sigma), 10nM heregulin-b1 (PeproTech), and 0.5µM forskolin (Sigma).

**Reprogramming of Vestibular Schwannomas:** between 0.2·10^6^-1·10^6^ of VS derived cells were reprogrammed using the CytoTune™-iPS 2.0 Sendai Reprogramming Kit (Thermo Fisher Scientific), a non-integrative cell reprogramming method holding the four Yamanaka factors (OCT4, SOX2, KLF4, and cMYC), according to manufacturer’s instructions.

**Cell culture Conditions**

**iPSC culture:** iPSCs were grown on growth factor-reduced Matrigel (BD Biosciences)-coated 6-well plates and cultured in mTESR Plus medium (STEMCELL Technologies). iPSCs were split using Accutase (Merk) and cells were seeded with Rock Inhibitor (STEMCELL Technologies) (1:1000) for 24h. When required, visibly differentiated cells were removed manually from the culture of *NF2*(-/-) cells. In this study, the *NF2*(+/+) FiPS line (FiPS Ctrl 1-SV4F-7, (FiPS Ctrl 1-SV4F-7 registered in the Spanish National Stem Cell Bank/ ESiO44C in https://hpscreg.eu/)) was used for all experiments as a control.

**Neural Crest (NC) differentiation:** differentiation of iPSC lines into NC was performed as previously described in Menendez et al. (2013) (10) with minor modifications (11). 9x10^4^ iPSCs were seeded on matrigel-coated 6-well plates in mTESR Plus medium. The following day, the medium was replaced with Neural Crest Differentiation Media (described below) and was replaced every day. NC were maintained in this medium and split with Accutase when necessary. After 10 days of differentiation, expression of NC specific markers was studied by flow cytometry.

**Neural Crest Differentiation Media**: DMEM:F12 (Gibco) 1:1; 5mg/mL BSA (Sigma); 500U/mL penicillin/ 500mg/mL streptomycin (Gibco); 2mM GlutaMAX (Gibco); 1x MEM non-essential amino acids (Gibco); 1x trace elements A; 1x trace elements B; 1x trace elements C (Corning); 2-mercaptoethanol (Gibco); 10 µg/mL transferrin (Sigma); 50µg/mL sodium L-ascorbate (Sigma); 10ng/mL heregulin-b1 (PeproTech); 200ng/mL LONG R3 IGFR (PeproTech); 8ng/mL basic fibroblast growth factor 2 (PeproTech), 2µM CHIR9902 (STEMCELL Technologies) and 20µM SB432542 (STEMCELL Technologies).

**Differentiation into SC in 2D:** to establish SC differentiation, 0.4x10^6^ NC cells/well were plated onto 0.1mg/mL poly-L-lysine (Sigma) and 4µg/mL laminin (Gibco) 6-well plates and cultured in SC differentiation media (SCDM) (described below) as previously described (11).

**Differentiation towards SC in 3D:** NC cells were detached with Accutase and, 2.25·10^6^ cells/well were seeded onto AggreWell TM800 24-well plates (Stem Cell Technologies) in 2mL SCDM (described below). The medium was changed twice a week removing 1 mL and replacing 1 mL of fresh SCDM. On days 7, 14 and 30 spheroids were collected and processed for subsequent analysis.

**Schwann Cell Differentiation Media (SCDM)**: DMEM: F12 (3:1); 500U/ml penicillin/ 500mg/mL streptomycin antibiotics (Gibco); 2mM GlutaMAX (Gibco), 5µM forskolin (Sigma); 50ng/mL heregulin-b1; 2% N2 supplement (Gibco); 1% FBS (Gibco). Medium was changed twice a week.

**Flow cytometry:** accutase-dissociated cells were resuspended in PBS-0.1% BSA and incubated with p75 primary antibody and subsequently incubated with Alexa Fluor 568 secondary antibody, followed by the incubation of Hnk1 primary antibody that was detected with Alexa Fluor 488-conjugated secondary antibody. Antibodies were incubated 30 min on ice. Flow cytometry analysis was performed using BD LSR Fortessa SORP and BD FACS Diva 6.2 software.

**iPSC characterization**

**Immunohistochemistry of pluripotency-associated markers:** iPSCs were fixed with 4% paraformaldehyde (PFA), blocked and permeabilized with TBS + 0.5% Triton X-100 + 6% donkey serum. Primary antibodies were incubated overnight in TBS + 0.1% Triton X-100 + 6% donkey serum. Secondary antibodies were incubated for 2h at 37ºC. Nuclei were stained with 4',6-diamino-2-fenilindol (DAPI). Antibodies are listed in Supplemental Table M1. Confocal images were taken using Leica TSC SPE/SP5 microscopes.

**Karyotype determination:** Karyotype of iPSCs was evaluated by G banded metaphase karyotype analysis, at the Hospital San Joan de Deu (Barcelona) and at the Cytogenetics Platform of the Haematology Department at Germans Trias I Pujol Hospital (Badalona).

**Alkaline Phosphatase activity**: Alkaline Phosphatase Blue Substrate Solution (Sigma) was used to demonstrate iPSC alkaline phosphatase activity. iPSC karyotype was assessed by treating cells with colcemid (Gibco) and processed as described (9).

RT-PCRs were performed to confirm the absence of the Sendai reprogramming virus and transgene expression (Supplemental Table M2).

**Merlin Western Blot:** Cells were lysed with RIPA buffer (50 mM Tris-HCl (pH 7.4), 150 mM NaCl, 1mM EDTA, 0.5% Igepal CA-630) supplemented with 3mM DTT (Roche), 1mM PMSF (Fluka), 1mM sodium orthovanadate (Sigma), 5mM NaF (Honeywell), 10 ug/ml leupeptin (Sigma), 5ug/ml aprotinin (Sigma) and 1xPhosSTOP (Roche). 50 µg of protein extracted from iPSC cell lines was loaded to SDS-PAGE (150V) and transferred onto PVDF membranes (1 hour 350 mA at 4^o^C). Odyssey Blocking Buffer TBS (LI-COR) was used to block the membranes. Primary antibodies were incubated at 4^o^C overnight. Membranes were later incubated with IRDye 680LT and IRDye 800CW secondary antibodies (1:1000 dilution, LI-COR) for 1h at room temperature and scanned and analysed using the Odyssey Infrared Imaging System (LI-COR). Western Blot primary antibodies used were α-NF2: NF2/Merlin antibody (ab88957) α-mouse (Abcam) to target merlin and α-vinculin [EPR8185] (ab129002) – α – rabbit (Abcam) was used to normalize protein expression between samples.

**iPSC differentiation into the three germinal layers through embryoid body (EB) formation:** iPSC colonies were lifted as usual with EDTA and transfer in a 96 well plate using a multichannel pipette in mTeSR-1 medium (Stem Cell Technologies). The 96 wells plate were centrifuged at 800g for 10 min and incubated at 37ºC and 5% CO2 for 24h. Then, early EBs were transferred to an ultra-low attachment plate in mTeSR-1 for additional 24h. After this time, EBs were transferred to matrigel-coated slide flasks and cultured in differentiation media for 21-28 days. Ectoderm medium: 50% Neurobasal medium, 50% DMEM/F12, 1% N2, 1% B27, 1% Glutamax and 1% Penicillin-Streptomycin; Endoderm medium: Knockout-DMEM, 10% FBS, 1% NEAA, 0.1% β-mercaptoethanol, 1% Glutamax and 1% Penicillin-Streptomycin (all Gibco); Mesoderm medium: Endoderm medium supplemented with 0.5mM ascorbic acid. Cells were analyzed by immunocytochemistry as described above. Antibodies are listed in Supplemental Table M1. Confocal images were taken using Leica TSC SPE/SP5 microscopes.

**iPSC direct differentiation to mesoderm.** Cells were seeded in a slide-flask with mTeSR medium. Twenty-four hours later (D0), mTeSR was changed by RPMI-B27 minus insulin medium with 8uM Chir. (RPMI-B27 media was composed by RPMI, GLX 1X, P/S 1X, NEEA 1X, B27(-/-) 1X and 2-mercaptoethanol 0,1X). On D1 medium was changed y RPMI-B27 with 2uM Chir. On D3: RPMI-B27 minus insulin with 5uM IWP4. D5: RPMI-B27 without insulin. D7: RPMI-B27 with insulin until D11.

**iPSC direct differentiation to endoderm.** Cells were seeded in matrigel-coated slide flasks with mTeSR medium until 80-90% of confluence and then stablish the protocol as follows:

- D1 - RPMI + P/S + GLX, add 100ng/ml activin A and 0,5uM Chir
- D2 - RPMI + P/S + GLX + 0,2% FBS, add 100ng/ml activi A and bFGF 0,1 µl/ml (100ugr/ml)
- D3 - RPMI + P/S + GLX + 2%FBS activin A + bFGF
- D4 - RPMI + P/S + GLX + B27 add FGF4 1µl/ml (500mM) + 3uM Chir
- D6-D8 – RPMI + P/S + GLX + B27 add FGF4 1µl/ml (500mM) + 3uM Chir

**Protocol for mix population (neurons and astrocytes)**. EBs were formed and seeded in Matrigel coated slide flasks as described above. The protocol followed:

- 7-10 days with NP-selection medium (Supplemental Table M3)
- 10-15 days NP-Expansion medium (Supplemental Table M3)
- Select and dissect structures neural-like
- Expansion in expansion medium (Supplemental Table M3)
- Seeded in Matrigel coated slide-flasks

Differentiation was analysed by immunocytochemistry as described above. Antibodies are listed in (Supplemental Table M1). Confocal images were taken using Leica TSC SPE/SP5 microscopes.

**Adhesion Assay:** 15.000 iPSCs were seeded in one well of a Matrigel-coated 96-well plate. Adhesion to the cell surface was observed after 24 hours and images were captured using LEICA DMIL6000 and LASAF software.

**iPSC Genomic and transcriptomic characterization**

**Tumor and iPSC genomic characterization:** genetic analysis was performed using the customized I2HCP panel as previously described (2) or Whole Exome Sequencing (WES). The latter was performed using KAPA HyperCap technology with KAPA HyperExome Probes (Roche) according to manufacturer’s instructions and sequenced in a NextSeq instrument (Illumina). Analysis of small nucleotide variants was performed with Mutect2 and annotated with Funcotator (3). Potential SNV and small indels were detected among the original cell lines and the paired CRISPR lines, GATK 4.2.6.1 was used for the entire process (3). To detect *NF2* splicing variants, tumors were studied both at RNA and DNA level. *NF2* variants were confirmed by Sanger Sequencing.

**SNP-array analysis**: SNP-array analysis was assessed using Illumina HumanOmniExpress v1 BeadChips (730,525 SNPs) according to manufacturers’ instructions. All samples were analyzed independently and treated as unpaired samples.

**Variant analysis:** Human Genome Variation Society (www.hgvs.org) nomenclature guidelines were used to name the mutation at the DNA level, its effect at the mRNA level, and the predicted resulting protein. The first nucleotide of the ATG translation initiation codon is denoted position þ1 according to the *NF2* mRNA sequence NM_000268.3 5. and NM_016418.

**CRISPR/Cas9 gene edition in iPSC lines:** CRISPR/Cas9 editing was performed with the ArciTect ribonucleoprotein (RNP) system (STEMCELL Technologies). The designed sgRNA targeted exon 2 of the *NF2* gene (GTACACAATCAAGGACACAG) using the Synthego - CRISPR Design Tools (<https://www.synthego.com/products/bioinformatics/crispr-design-tool>). TransIT-X2® Dynamic Delivery System (Mirus) was used for transfection according to manufacturers’ instructions.

***NF2* gene characterization after CRISPR/Cas gene editing:**  *NF2* exon 2 was screened by Sanger sequencing in each single iPSC edited cell clone. For each cell clone with an identified *NF2* pathogenic variant, NF2 gene cDNA coding sequence, containing the variant at exon 2 and the variant identified previously in the tumor, was cloned using the Gateway® Gene Cloning system (Invitrogen) and analysed by Sanger sequencing to determine if both variants were located at the same allele (*cis*) or in different alleles (*trans*).

**Methods for SNV and indel off target analysis:** Materials including genome, indel and SNP databases were retrieved from ftp.broadinstitute.org at /bundle/b37. For alignment, bwa 0.7.17 (4) was used under hg19 genome assembly. Following GATK Best Practices (3), aligned bam files were marked for duplicated reads and Base Quality Score Recalibration was applied to each specimen. Afterwards, Mutect2 with orientation bias correction was used for SNV and indel calling for each pair pre/post CRISPR cell line call. Variants with TLOD > 20, counts > 30 and AF > 30% were considered as a potential CRISPR off-target candidates. Conflictive and suspicious calls were manually reviewed using IGV (5). For potential CRISPR off-target sites created from our Cas sequence, we used cas-offinder online version (<http://www.rgenome.net/cas-offinder/>) (6) with lenient thresholds (max DNA/RNA Bulge Size 2 and max mismatch of 3). These sites were flanked +/-1000bp for subsequent analysis. Pybedtools (7) the Python wrapper for Bedtools (8) was used to intersect the detected SNP/indels sites with the potential off target sites. We consider at least 1bp of overlap as an off-target. In order to annotate the variants, GATK’s Funcotator with source version 1.7.20200521s was used.

**NC and SC cellular characterization**

**Proliferation Assay (Click-iT Edu assay):** Cells were plated on matrigel-coated 6-well plates, and feeded daily with Neural Crest Differentiation Media. After 72h cells were treated with 20 µM EdU for 2 hours and processed using Click-iT Plus EdU Flow Cytometry Assay Kits (Thermo Fisher) according to manufacturer protocol. Cells were also stained with DAPI to detect DNA content. Data was collected and analyzed using an BD LSR Fortessa SORP and BD FACSDiva 6.2 software.

**Scratch assay:** Cells were plated onto Matrigel-coated 6-well plates. After reaching 80% of confluence, a gap was created using a pipette tip and the migration capacity of the cells was measured by taking images of the same region 6 and 24 hours after the scratch was created. Images were captured using LEICA DMIL6000 and LASAF software.

**Immunocytochemistry:** cells or spheroids were fixed in 4% paraformaldehyde in PBS for 15min at room temperature (RT), permeabilized with 0.1%Triton-X 100 in PBS for 10 min at RT, blocked in 10% FBS in PBS for 15 min at RT, and incubated with the indicated antibodies (**Supplemental Table M1**) overnight at 4°C. Secondary antibodies Alexa Fluor 488 and Alexa Fluor 568 were incubated for 1h at RT. Nuclei were stained with DAPI and images captured using LEICA DMIL6000 and LASAF software.

**Transcriptomic analysis**

**RNA processing, sequencing and analysis:** Total RNA extraction from iPSCs, NC cells and SC-differentiating spheroids was extracted with the 16 LEV simplyRNA Purification kit (Promega), following manufacturer’s instructions. RNA was quantified with a Nanodrop 1000 spectrophotometer (Thermo Scientific). The polyA RNA libraries were sequenced on an Illumina Novaseq 6000 in 150 bp pair-end mode. For RNAseq processing, genome GRCh37.p13 and gene annotation gencode version 19 were used. Reads were aligned with STAR version 2.7.10a (12) and count files were produced with QoRTs version 1.3.6 (13). These raw counts were used for DESeq2 differential expression (DE) analysis with Wald significance test and Benjamini & Hochberg correction (FDR) correction. A corrected p-value of 0.01 and an absolute Log2-fold change of 1 was used for considering a gene as DE. Volcano plots were performed using EnhancedVolcano (with the <https://github.com/kevinblighe/EnhancedVolcano>) outputs from Deseq2. To account for more direct measurement, barplots in Figure 1F were instead normalized by Trimmed Mean of M-values from EdgeR (14) and then RPKMs were calculated for every gene. The *vst* normalization from DESeq2 (15) was used for unsupervised cluster heatmaps with Euclidean metric and Ward method. PCA features were chosen using the top 2000 genes with the highest SD over vst normalization. Pathway enrichment analyses for DE genes were performed using GSEA pre-ranked test (16) using the “stat” value from Deseq2. A FDR <0.05 was considered as significant. Single sample GSEA (ssGSEA) with MSigDB version 2022.1.Hs (17) was used when comparing more than two groups of samples. Enrichr (18,19) was used only with DE genes as input.

**ACKNOWLEDGEMENTS**

We thank patients, families and the NF patients’ associations (AcNeFi, Chromo 22 and AANF) that support to this project, the CSUR of Phakomatoses and the HGTP Clinical Services and staff for their collaboration. We thank the IGTP Flow Cytometry and IGTP Genomics core facilities and their staff for their contribution and technical support.

**CONFLICT OF INTEREST STATEMENT**

The authors declare no competing interests

**DATA AVAILABILITY STATEMENT**

The authors confirm that the data supporting the findings of this study are available within the article [and/or] its supplementary materials.

# **SUPPLEMENTAL REFERENCES**

1. Baser ME, Friedman JM, Wallace AJ, Ramsden RT, Joe H, Evans DGR. Evaluation of clinical diagnostic criteria for neurofibromatosis 2. Neurology. 2002 Dec;59(11):1759–65.

2. Castellanos E, Gel B, Rosas I, Tornero E, Santín S, Pluvinet R, et al. A comprehensive custom panel design for routine hereditary cancer testing: Preserving control, improving diagnostics and revealing a complex variation landscape. Sci Rep. 2017;7.

3. Van der Auwera GA, O’Connor BD. Genomics in the Cloud: Using Docker, GATK, and WDL in Terra [Internet]. O’Reilly Media; 2020. Available from: https://books.google.es/books?id=vsXaDwAAQBAJ

4. Li H, Durbin R. Fast and accurate short read alignment with Burrows-Wheeler transform. Bioinformatics. 2009;25(14):1754–60.

5. Robinson JT, Thorvaldsdóttir H, Winckler W, Guttman M, Lander ES, Getz G, et al. Integrative genomics viewer. Vol. 29, Nature Biotechnology. 2011. p. 24–6.

6. Bae S, Park J, Kim JS. Cas-OFFinder: A fast and versatile algorithm that searches for potential off-target sites of Cas9 RNA-guided endonucleases. Bioinformatics. 2014;30(10):1473–5.

7. Dale RK, Pedersen BS, Quinlan AR. Pybedtools: A flexible Python library for manipulating genomic datasets and annotations. Bioinformatics. 2011;27(24):3423–4.

8. Quinlan AR, Hall IM. BEDTools: A flexible suite of utilities for comparing genomic features. Bioinformatics. 2010;26(6):841–2.

9. Campos PB, Sartore RC, Abdalla SN, Rehen SK. Chromosomal spread preparation of human embryonic stem cells for karyotyping. Journal of Visualized Experiments. 2009;(31):4–7.

10. Menendez L, Kulik MJ, Page AT, Park SS, Lauderdale JD, Cunningham ML, et al. Directed differentiation of human pluripotent cells to neural crest stem cells. Nat Protoc. 2013 Jan;8(1):203–12.

11. Carrió M, Mazuelas H, Richaud-Patin Y, Gel B, Terribas E, Rosas I, et al. Reprogramming Captures the Genetic and Tumorigenic Properties of Neurofibromatosis Type 1 Plexiform Neurofibromas. Stem Cell Reports. 2019;12.

12. Dobin A, Davis CA, Schlesinger F, Drenkow J, Zaleski C, Jha S, et al. STAR: ultrafast universal RNA-seq aligner. Bioinformatics. 2013 Jan;29(1):15–21.

13. Hartley SW, Mullikin JC. QoRTs: a comprehensive toolset for quality control and data processing of RNA-Seq  experiments. BMC Bioinformatics. 2015 Jul;16(1):224.

14. Robinson MD, McCarthy DJ, Smyth GK. edgeR: a Bioconductor package for differential expression analysis of digital  gene expression data. Bioinformatics. 2010 Jan;26(1):139–40.

15. Love MI, Huber W, Anders S. Moderated estimation of fold change and dispersion for RNA-seq data with DESeq2. Genome Biol. 2014;15(12):550.

16. Subramanian A, Tamayo P, Mootha VK, Mukherjee S, Ebert BL, Gillette MA, et al. Gene set enrichment analysis: A knowledge-based approach for interpreting genome-wide expression profiles [Internet]. 2005. Available from: www.pnas.orgcgidoi10.1073pnas.0506580102

17. Barbie DA, Tamayo P, Boehm JS, Kim SY, Moody SE, Dunn IF, et al. Systematic RNA interference reveals that oncogenic KRAS-driven cancers require TBK1. Nature. 2009 Nov 5;462(7269):108–12.

18. Kuleshov M V., Jones MR, Rouillard AD, Fernandez NF, Duan Q, Wang Z, et al. Enrichr: a comprehensive gene set enrichment analysis web server 2016 update. Nucleic Acids Res. 2016 Jul 8;44(1):W90–7.

19. Prakash YS, Singh A, Shannon CP, Kim YW, Demarco ML, Gauvreau GM, et al. Identifying Molecular Mechanisms of the Late-Phase Asthmatic Response by Integrating Cellular, Gene, and Metabolite Levels in Blood Mucins and Their Sugars Critical Mediators of Hyperreactivity and Inflammation [Internet]. Vol. 13, Ann Am Thorac Soc. 2016. Available from: www.atsjournals.org.www.atsjournals.org.

# **MATERIAL AND METHODS SUPPLEMENTAL TABLES**

| **Supplemental Table M1. List of antibodies.** | | | | | | | | | | | | | | | | | | | | |
| --- | --- | --- | --- | --- | --- | --- | --- | --- | --- | --- | --- | --- | --- | --- | --- | --- | --- | --- | --- | --- |
| **Antibody** | Rabbit IgG anti-NF2 / Merlin | Rabbit IgG anti-Vinculin | Mouse IgG anti-OCT3/4 | Mouse IgG anti OCT4 (OCT3) | Rabbit IgG anti-SOX2 | Goat IgG anti-NANOG | Rat IgM anti-SSEA3 | Mouse IgG anti-SSEA4 | Mouse IgM anti TRA-1-81 | Goat IgG anti-FOXA2 | Rabbit IgG anti-GATA4 | Mouse IgG anti SMA | Mouse IgM anti-ASA | Rabbit IgG anti GFAP | Mouse IgG anti-TUJ1 | Mouse IgG anti [NGFR5] to p75 NGF Receptor | Rabbit IgG anti-S100B | Mouse IgG anti-AP2 | Rabbit IgG anti-Sox10 | Mouse IgG anti-HNK1 |
| **Supplier and Reference** | Abcam, ab109244 | Abcam, ab129002 | Santa Cruz Biotechnology, Sc-5279 | Stem Cell Technologies, #60059 | Pierce Antibodies, PA1-16968 | R&D Systems, AF1997 | Hybridoma Bank, MC-631 | Hybridoma Bank, MC-813-70 | Millipore, MAB4381 | R&D Systems, AF2400 | Santa Cruz Biotechnology, Sc-9053 | Sigma, A5228 | A2172 | Dako, Z0334 | Bio Legend, MMS-435P | Abcam, ab3125 | Dako, Z0311 | MA1-872 | Abcam, ab155279 | SIGMA, C6680 |
| **Dilution** | 1:200 | 1:1000 | 1:60 | 1:100 | 1:100 | 1:25 | 1:3 | 1:3 | 1:400 | 1:50 | 1:50 | 1:400 | 1:400 | 1:500 | 1:500 | 1:100 (IF)  1:1000 (FACS) | 1:1000 | 1:50 | 1:50 | 1:1000 (FACS) |

| **Supplemental Table M2. List of primers for RT-PCR to detect SeV genome and transgenes set** | | | |
| --- | --- | --- | --- |
| **Target** |  | **Primer** | **Product size (bp)** |
| SeV | *Forward* | GGATCACTAGGTGATATCGAGC | 181 |
|  | *Reverse* | ACCAGACAAGAGTTTAAGAGATATGTATC |  |
| KOS | *Forward* | ATGCACCGCTACGACGTGAGCGC | 528 |
|  | *Reverse* | ACCTTGACAATCCTGATGTGG |  |
| Klf4 | *Forward* | TTCCTGCATGCCAGAGGAGCCC | 410 |
|  | *Reverse* | AATGTATCGAAGGTGCTCAA |  |
| L-Myc | *Forward* | GAGAAGAGGATGGCTACAGAGA | 237 |
|  | *Reverse* | GACGTGCAACTGTGCTATCT |  |

| **Supplemental Table M3. Embryoid Body (EB) direct differentiation media** | | |
| --- | --- | --- |
| **Selection medium (200ml)** | **Expansion medium (200ml)** | **Differentiation medium (200ml)** |
| DMEM-F12 192.8ml | DMEM-F12 191.76ml | DMEM-F12 96.5ml |
| P/S 2ml | P/S 2ml | Neurobasal medium 96.5ml |
| GLX 2ml | GLX 2ml | P/S (1%) 2ml |
| b-mercaptoethanol 200µl | b-mercaptoethanol 200µl | GLX (1%) 2ml |
| NEAA 2ml | NEAA 2 ml | N2 (0.5%) 1ml |
| N2 (0.5%) 1ml | N2 (1%) 2ml | B27 (1%) 2ml |
|  | hFGF 40µl (20ng/ml) |  |

# **SUPPLEMENTAL FIGURES**

**Figure S1**.***NF2* cDNA gene characterization after CRISPR/Cas gene editing.** Sanger sequencing of the generated and selected clons. The following LOF variants are displayed in cloned cDNA: VSi-267 (c.784C>T), FiPs-CasB2 (c.212_213delCA), VSi-25 (c.1736A>G, r.1585_1747del), VSi-267-CasD2 (c.784C>T, c.212_213del), FiPs-CasH6 (c.211dup), VSi-25-CasD7 (c1736A>G, r.1585_1747del; c.211dup), while FiPs-CasH6 and VSI-25-CasD7 (c.211dup) were sequencend directly from the iPSC cell culture.


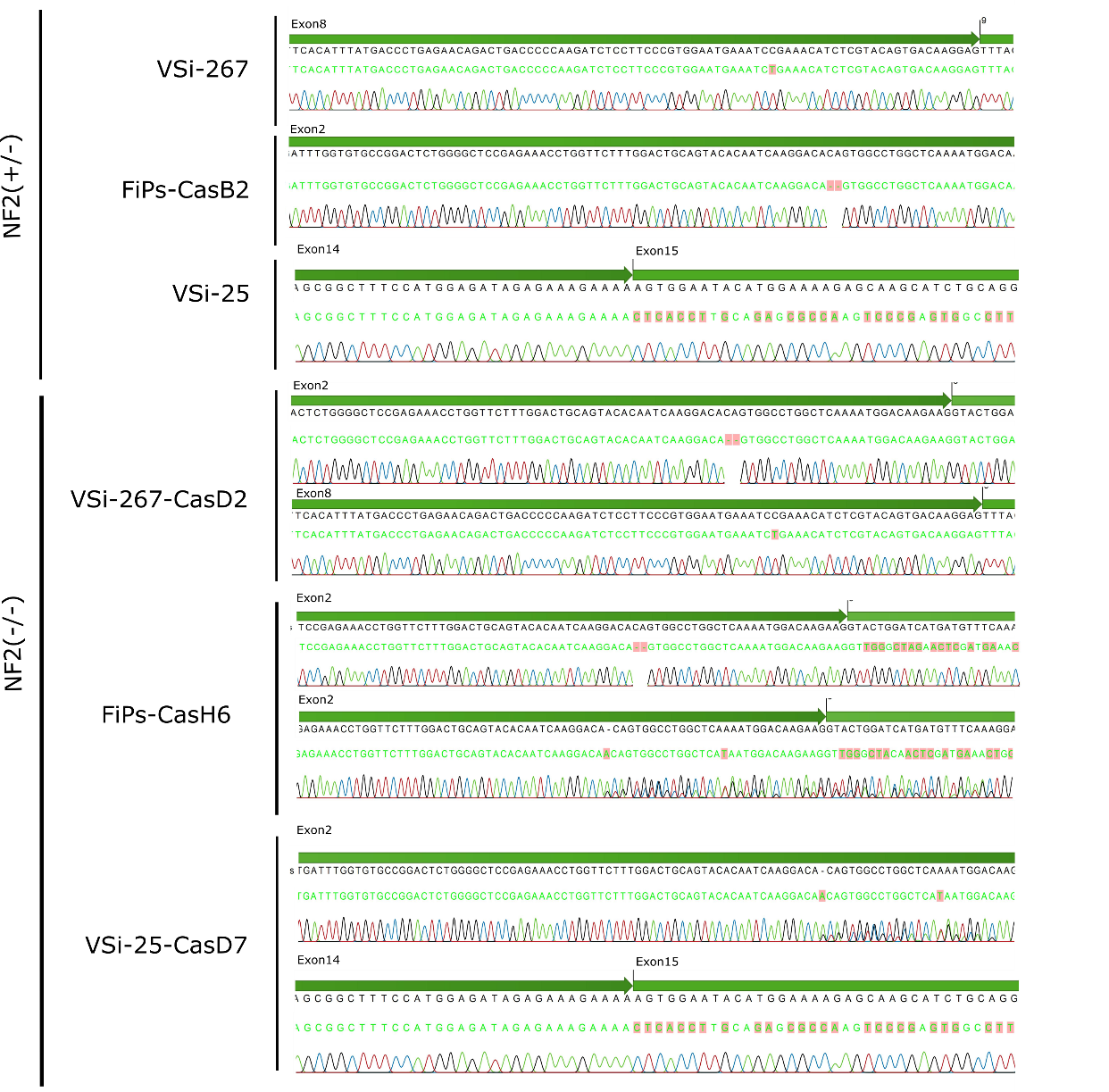


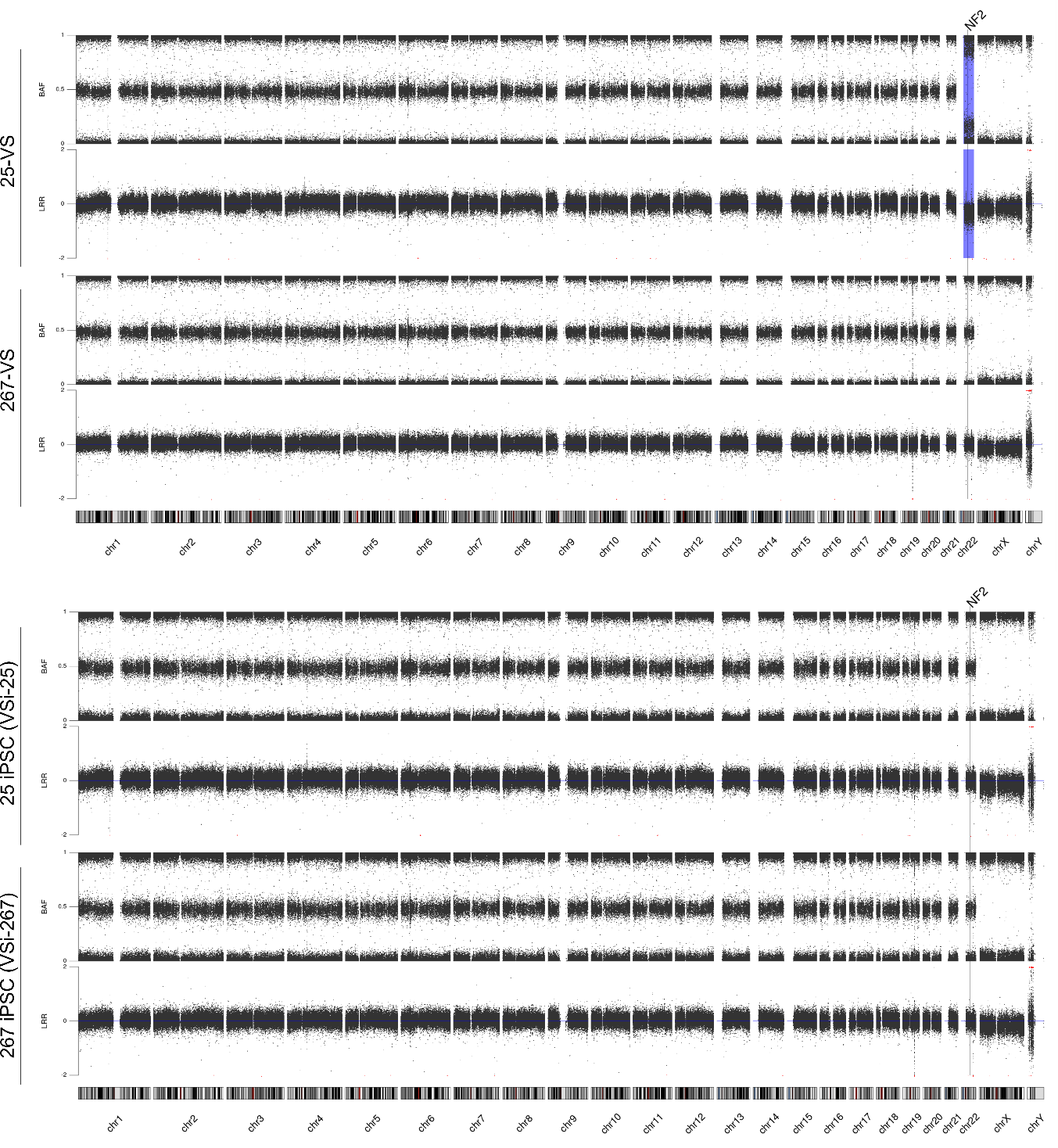
**Figure S2 –** SNP-array analysis showed loss of heterozygosity (LOH) on chromosome 22q in VS-25 and no presence of complex rearrangements. VSi-25 and VSi-267 iPSCs SNP-array analysis showed no differences with respect to the tumor of origin, with the exception for VS-25, in which the LOH on chromosome 22q as the second hit was not present in the (+/-) clones obtained from this tumor. BAF: B allele frequency; LRR: Log R Ratio; The blue shaded region denotes the absence of *NF2* due to the LOH.

**Figure S3.** **Characterization of iPSC clones.** (A) Immunochemistry of pluripotency markers NANOG, OCT4, and SOX2 (in green), TRA-1-81, SSEA3 (in red); Scale bar, 75µM; Cell nuclei were stained with DAPI; (B) Alkaline Phosphatase (ALP) Staining; (C) Karyotype of lines at passage 20 (46, XY); (D) Immunochemistry to demonstrate the capacity of the lines to differentiate *in vitro* to the three primary germ layers of *NF2*(+/-) lines: mesoderm (ASMA in green and ASA in red), ectoderm (TUJ1 in green and GFAP in red) and endoderm (AFP in green and FOXA2 in red). For *NF2*(-/-), a directed differentiation was performed due to the inability of these lines to generate EBs, for these: mesoderm (ASMA in green and GATA4 in red), ectoderm TUJ1 in green, GFAP in red and PAX6 in pink) and endoderm (AFP in green, FOXA2 and SOX17 in red). Scale bar, 75µm. (E) Adhesion capacity of the iPSC lines. Cells were detached using accutase and re-plated in Matrigel-coated plates. After 24h, micrographs were taken to visualize adhesion capacity. Scale bar, 250µm. The adhesion capacity of *NF2*(-/-) clones appeared to be inferior to that of *NF2*(+/+) or *NF2*(+/-) in iPSCs under these culture conditions.


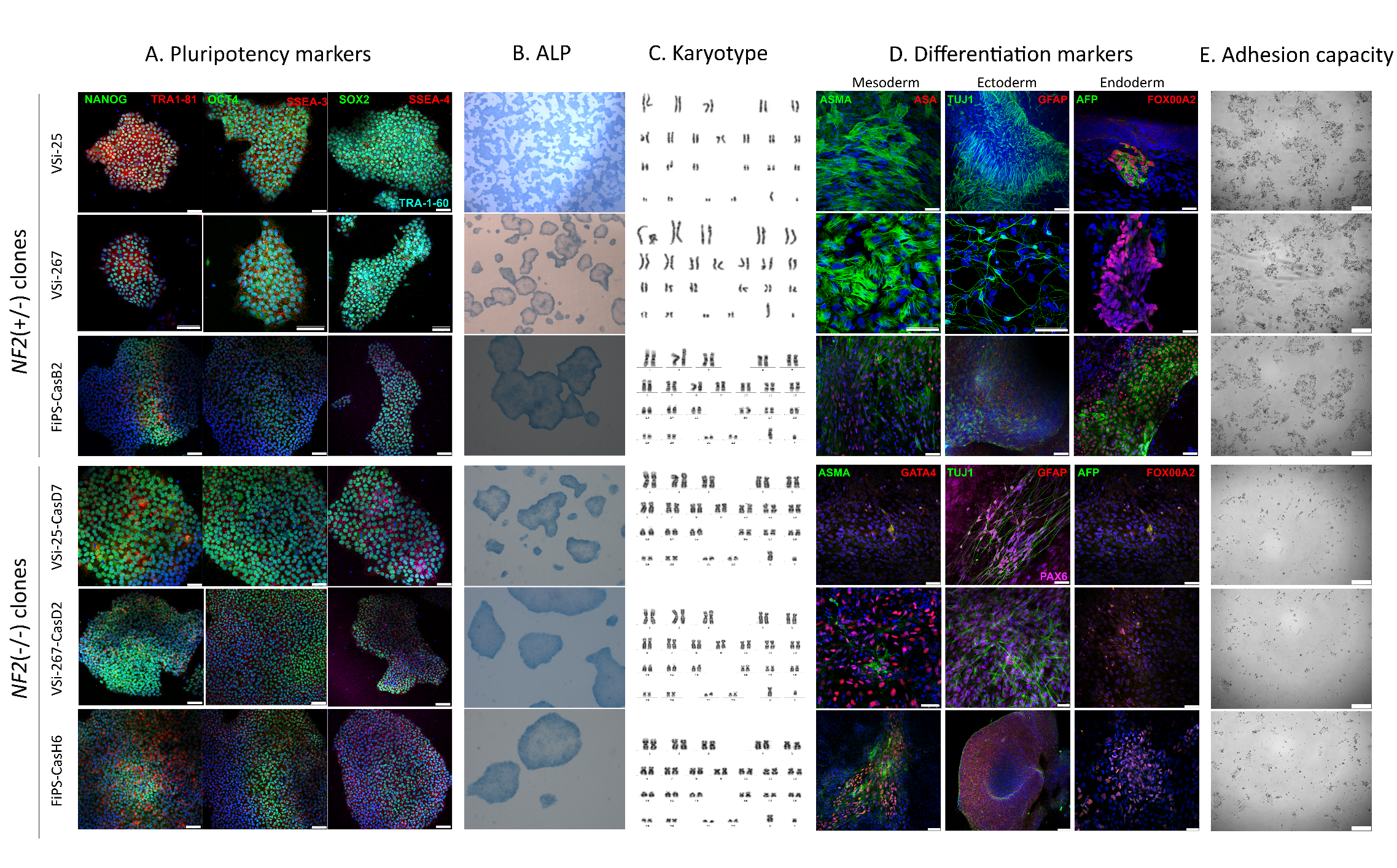


**Figure S4.** **Neural Crest Characterization.** (A) NC Morphology of *NF2*(+/-) and *NF2*(-/-) cell lines; Scale bar, 75µm; (B) Flow cytometry assays of the NC markers NGFR (p75) and Hnk1. The percentage of p75 and Hnk1-positive cells is shown in white. P4 and P8 stand for passages 4 and 8, respectively; (C) Immunocytochemistry of AP2 (green), p75 (green) and S100B (red), Scale bars, 25µm; Oct4 (green) and SOX10 (red), Scale bar, 100µm; DAPI (blue) was used to stain cell nuclei. (D) Scratch assay. Scale bar, 75µm; (E) Proliferation assay. Bars express the mean ±SD percentage of EdU Positive cells from three independent experiments in the *NF2*(+/+) cells and the mean of the three lines for the *NF2*(+/-) and *NF2*(-/-) genotypes. Mann-Whitnney U statistical test was performed among groups and showed no statistically significant differences.


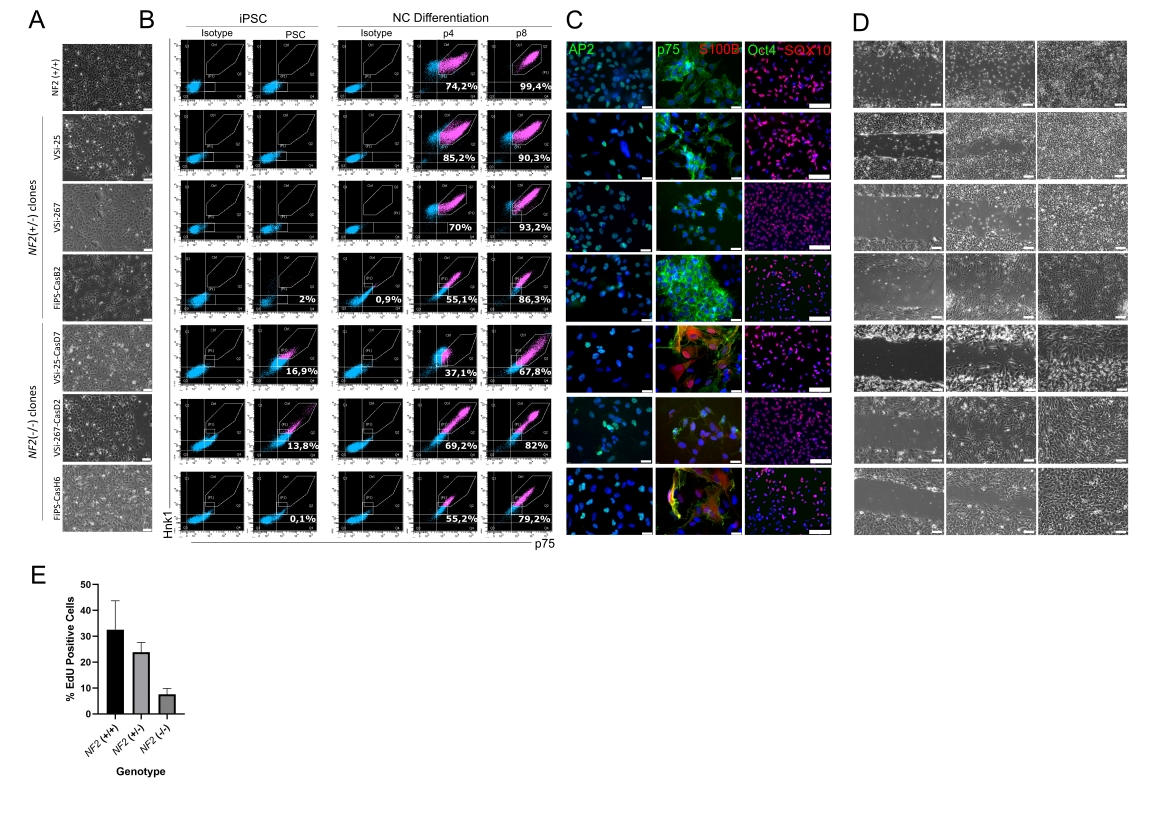


**Figure S5** –**Phase-contrast images of *NF2* (+/+), *NF2* (+/-) and *NF2* (-/-) cell lines during SC differentiation**. *NF2* deficient cells did not show capacity to maintain attachment to the cell culture already after 5 days under the SC differentiation media. Scale bar, 75µM.


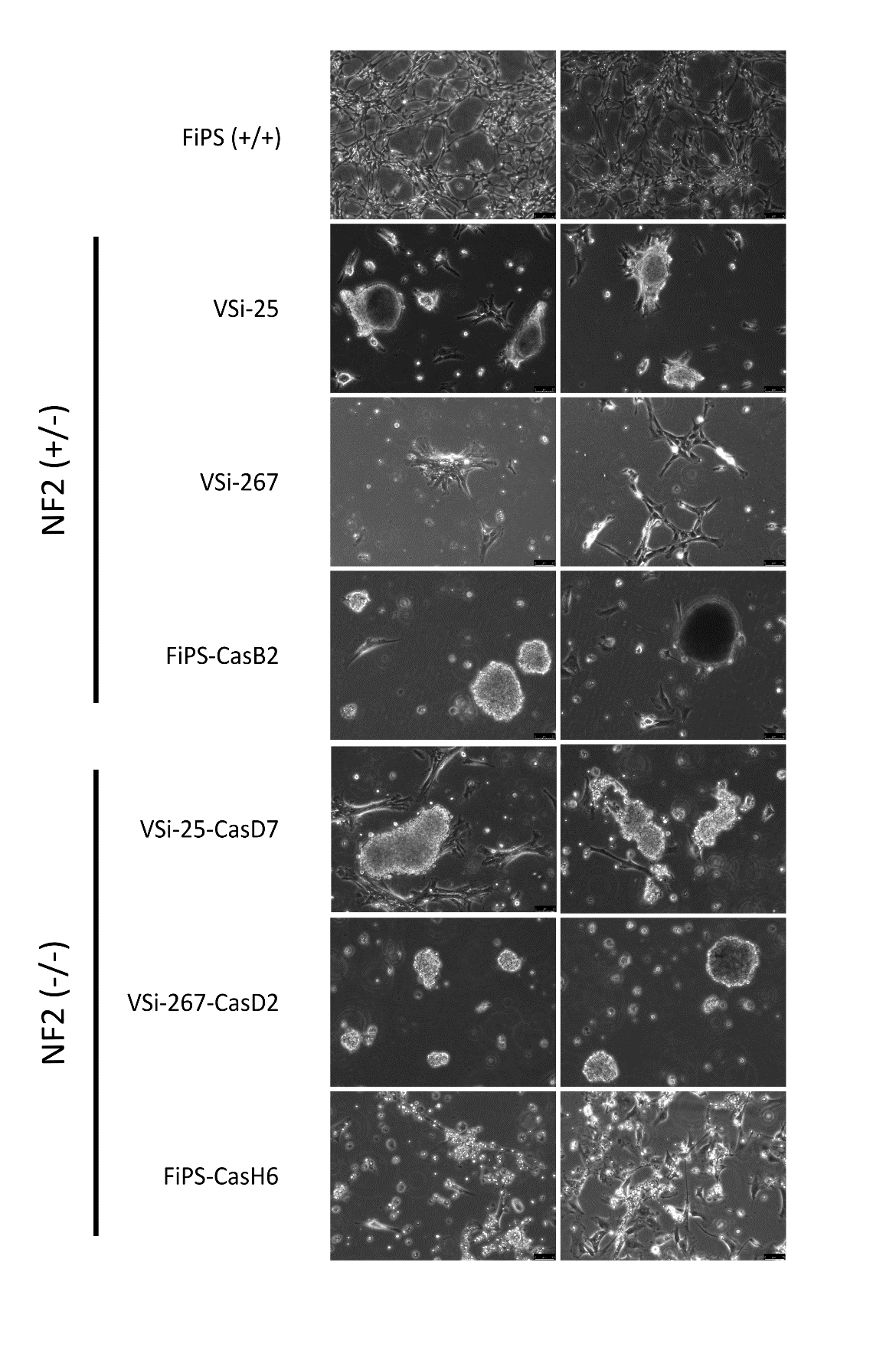


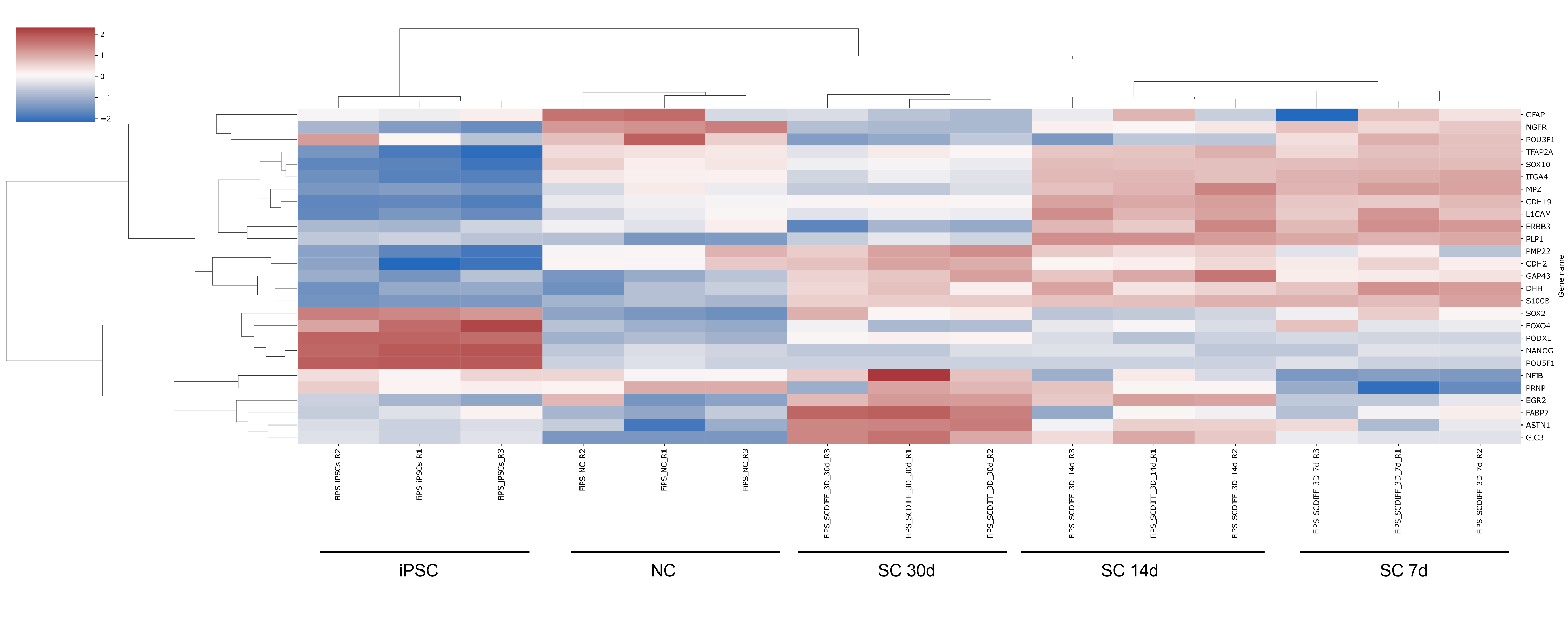
**Figure S6**. Heatmap of the control *NF2*(+/+) line (FiPS) in the 3D differentiation protocol, representing expression of genes expressed over the stages of the differentiation protocol. Data shown is from three independent differentiation experiments. 7d, 14d and 30d stand for 7, 14 and 30 days under SC differentiation conditions, respectively.


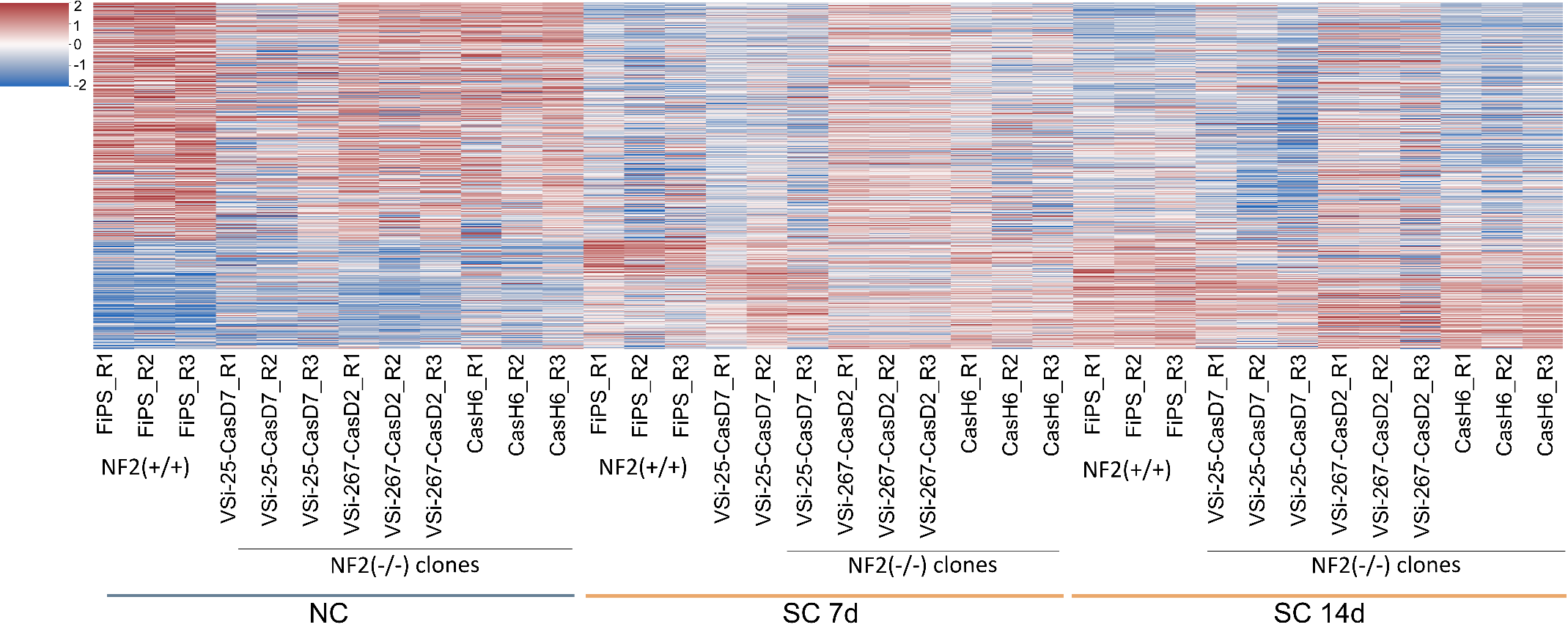
**Figure S7**. *In vitro* NC-SC expression roadmap of the control line (FiPS, *NF2*(+/+)) in the 2D differentiation protocol and the *NF2*(-/-) lines in the 3D differentiation protocol. Data from three independent differentiation experiments is shown (R1, R2 and R3). 7d and 14d stand for 7 and 14 days under SC differentiation conditions, respectively.

**Figure S8**. **Schwann Cell Differentiation in 3D cultures** (A) PCA analysis over the 2000 genes with highest SD. Components 1 and 3 are shown, with the explained variance indicated for each of them. Also, all SCs differentiating in 3D clustered together independently of the genotype or days of differentiation (B) Immunochemistry of p75 (green) and S100B (red) after 14 days of SC differentiation, Scale bar, 250µm. (C) Gene expression at day 14 of the 3D SC differentiation protocol. Bars express mean normalized expression ±SD from three independent experiments.


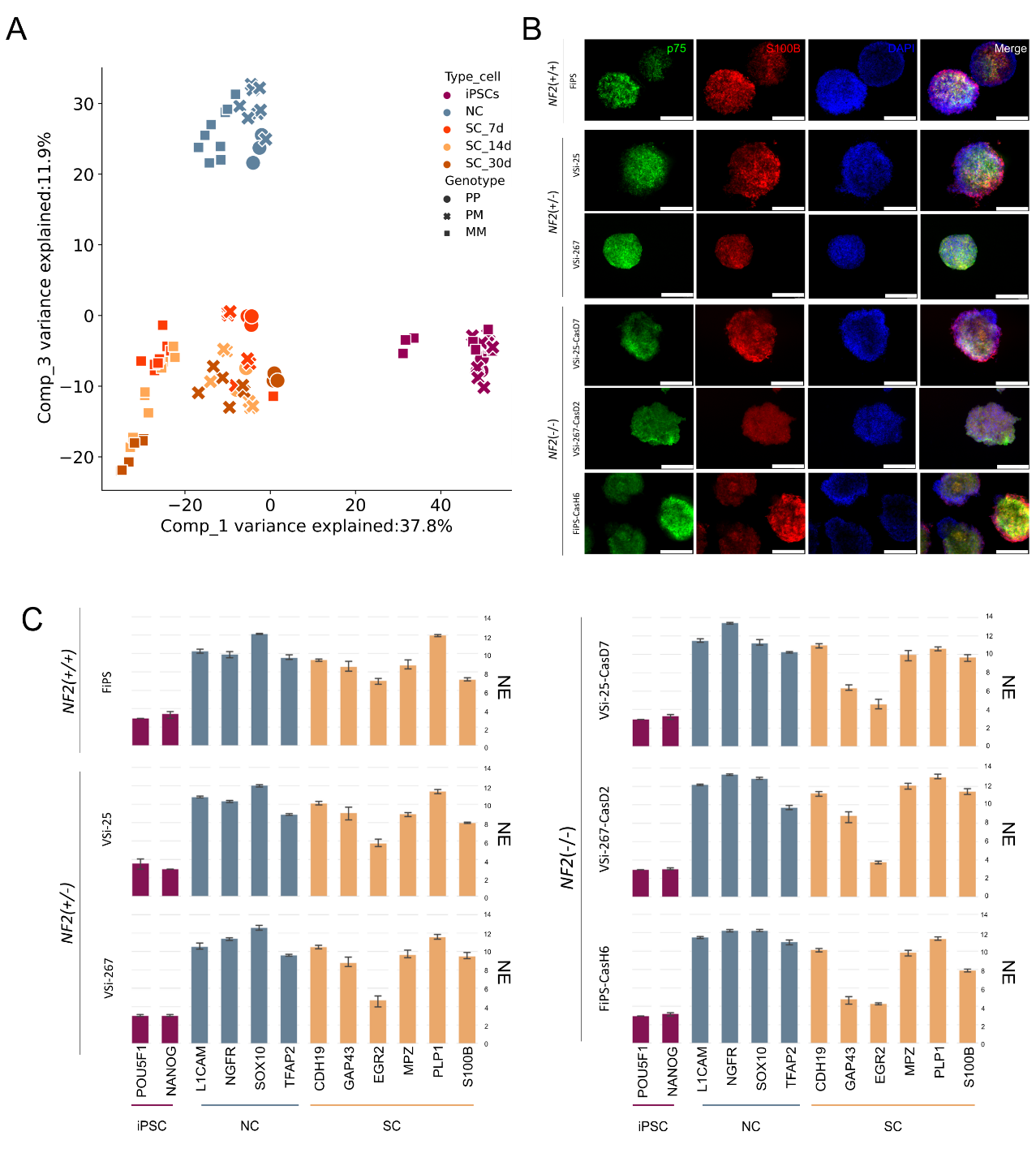


**Figure S9**. **Gene expression analysis of SC-like spheroids.** (A) Volcano plot for DE genes and expressed pseudogenes between NF2(+/-) and NF2(+/+). X-axis shows Log2-fold changes among conditions. Y-axis represents the p-value in –log10 scale; padj < 0.01 logfc =1 (B) GSEA analysis between NF2(+/-) and NF2(+/+) are shown. Only Hallmark pathways with FDR<0.05 are displayed. Green bars on X-axis account for the enrichment score on each of them. (C) Volcano plot for DE genes and expressed pseudogenes between NF2(+/+) vs NF2(-/-). (D) GSEA analysis between NF2(+/+) vs NF2(-/-). (E) Volcano plot for DE genes and expressed pseudogenes between NF2(+/-) and NF2(-/-). (F) GSEA analysis between NF2(+/-) vs NF2(-/-).


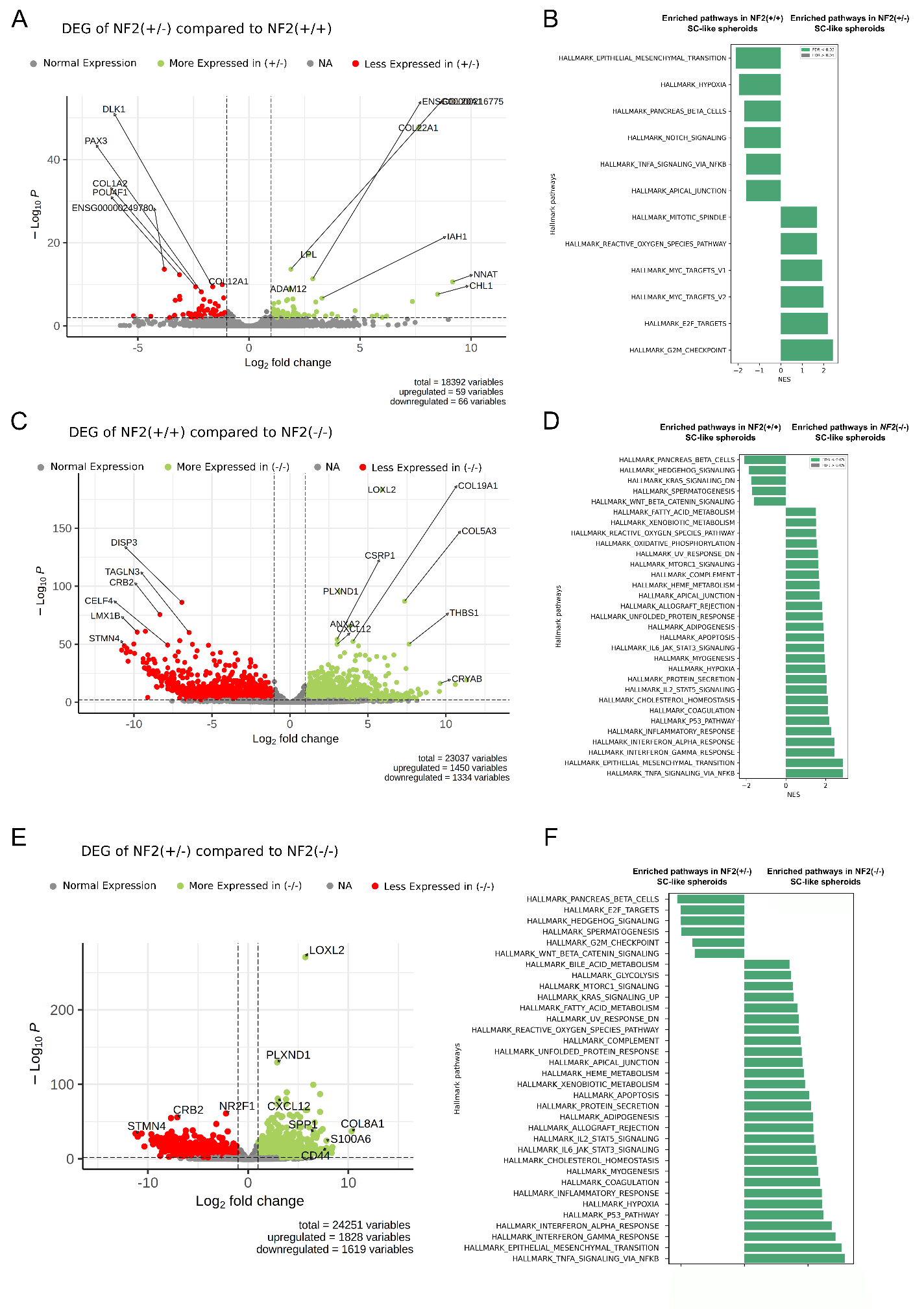

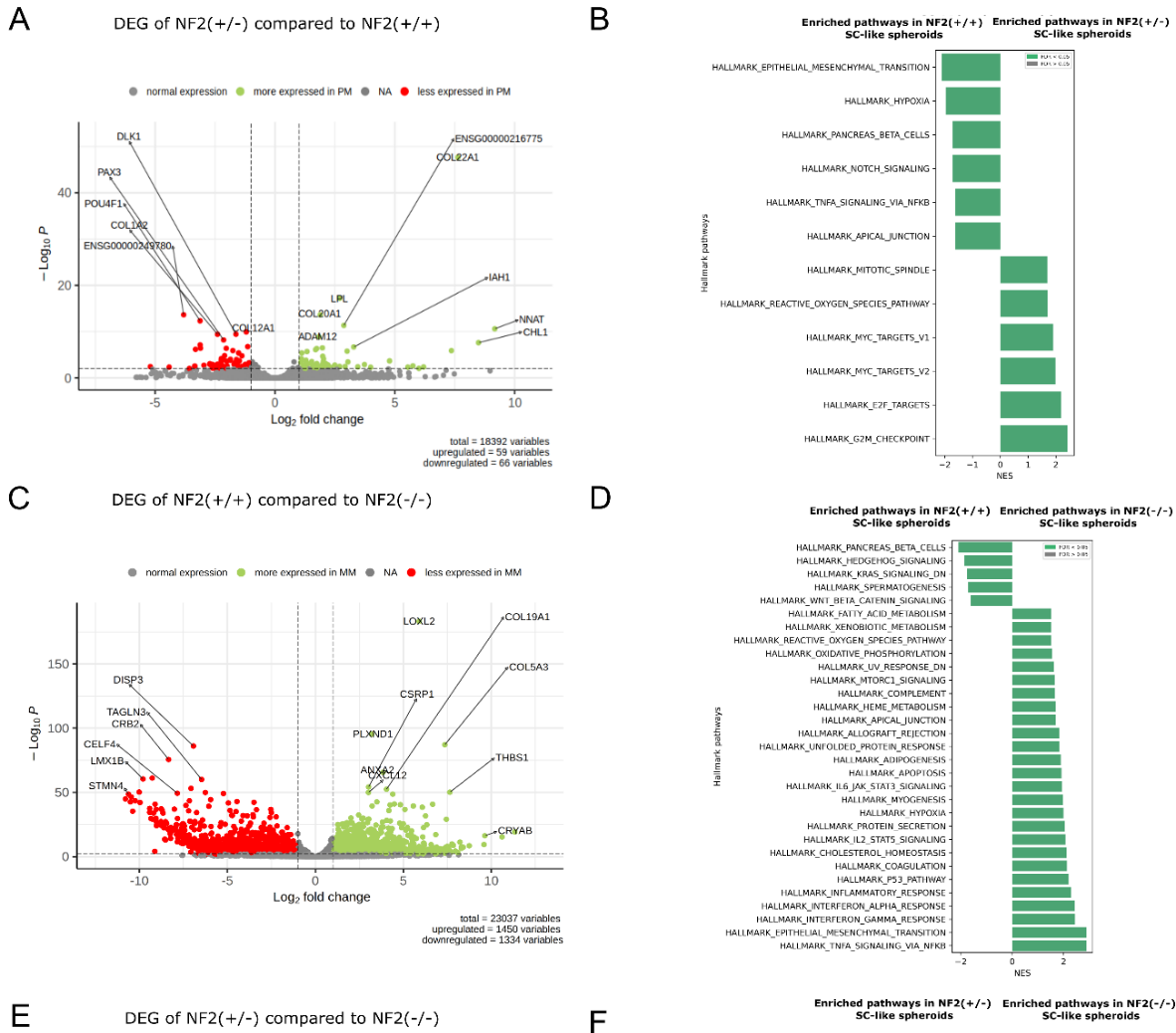


**Figure S10**. Gene expression analysis with vst values are shown for each gene. Bars express mean normalized expression ±SD from three independent experiments. T-test was performed for each individual comparison among genotypes. NE stands for Normalized Expression. Mean and standard deviation are shown. Significant comparisons are shown as *<0.05; **<0.01, ***<0.001.


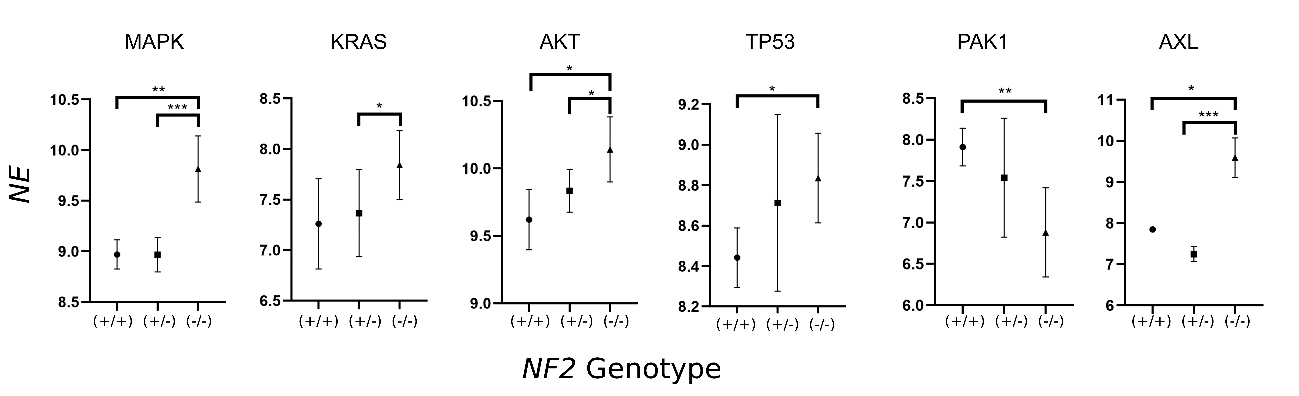


# **SUPPLEMENTAL TABLES**

| Supplemental Table S1. | | |
| --- | --- | --- |
| Patient ID | 25 | 267 |
| Sex | XY | XY |
| Diagnostic | NF2 | NF2 |
| Age at diagnosis | 47 | 14 |
| Tumor load | BVS | BVS, Peripheral SC and multiple intraspinal SC |
| Number of interventions | 2 | 3 |
| Age at first intervention | 47 | 14 |
| **VS ID** | VS-25 | VS-267 |
| ***NF2* Germline Mutation** | g.83045A>G | g.62758C>T |
| ***NF2* Somatic Mutation** | LOH | g.73394G>A |

**Table S2. iPSCs lines information**

| **Table S3. VSs Reprogramming information** | | | | | | |
| --- | --- | --- | --- | --- | --- | --- |
|  | | | | **iPSCs clones genotype** | | |
| **Patient ID** | **VS ID** | **Method** | **Number of Clones Analyzed** | ***NF2*(+/+)** | ***NF2*(+/-)** | ***NF2*(-/-)** |
| 25 | VS-25 | SeV | 38 | 0 | 38 | 0 |
| 267 | VS-267 | SeV | 12 | 0 | 12 | 0 |

| **Table S4. WES analysis of the CRISPR-generated lines** | | | | | | | | | | | | | | |
| --- | --- | --- | --- | --- | --- | --- | --- | --- | --- | --- | --- | --- | --- | --- |
| **FiPS-CasB2** | | | | | | | | | | | | | | |
| **Gene Symbol** | | **Variant Classification** | | **Variant Type** | | **Genome Change** | | | **cDNA Change** | | **Protein Change** | | **SwissProt acc_Id** | |
| DNAJC10 | | Missense | | SNP | | chr2:183582950C>A | | | c.137C>A | | p.A46E | | Q8IXB1 | |
| LIMD1 | | Missense | | SNP | | chr3:45637329G>A | | | c.958G>A | | p.G320S | | Q9UGP4 | |
| HMGXB3 | | Intronic | | SNP | | chr5:149425299T>A | | | c.e16+51T>A | |  | | Q12766 | |
| GBA2 | | Missense | | SNP | | chr9:35748662G>A | | | c.40C>T | | p.P14S | | Q9HCG7 | |
| B3GNTL1 | | Intronic | | DEL | | chr17:80993071_80993073delTCG | | | c.e10+95CGAA>A | |  | | Q67FW5 | |
| KANK3 | | Missense | | SNP | | chr19:8400524G>A | | | c.187C>T | | p.R63C | | Q6NY19 | |
| NF2 | | Frameshift | | DEL | | chr22:30032834_30032835delAC | | | c.209_210delAC | | p.T71fs | | P35240 | |
|  | | | | | | | | | | | | | | |
| **FiPS-CasH6** | | | | | | | | | | | | | | |
| **Gene Symbol** | | **Variant Classification** | | **Variant Type** | | **Genome Change** | | | **cDNA Change** | | **Protein Change** | | **SwissProt acc_Id** | |
| YRDC | | Missense | | SNP | | chr1:38272621C>T | | | c.532G>A | | p.A178T | | Q86U90 | |
| EIF2B3 | | Intronic | | SNP | | chr1:45443916T>C | | | c.e10-71A>G | |  | | Q9NR50 | |
| CRNN | | Intronic | | SNP | | chr1:152384514C>A | | | c.e2-58G>T | |  | | Q9UBG3 | |
| OR10J1 | | Missense | | SNP | | chr1:159409651C>A | | | c.70C>A | | p.Q24K | | P30954 | |
| BLOC1S4 | | Silent | | SNP | | chr4:6718341C>A | | | c.405C>A | | p.I135I | | Q9NUP1 | |
| THAP6 | | 5'UTR | | SNP | | chr4:76441986C>A | | |  | |  | | Q8TBB0 | |
| CHD1 | | Frameshift | | DEL | | chr5:98236745delT | | | c.629delA | | p.K210fs | | O14646 | |
| MTCH1 | | Intronic | | SNP | | chr6:36944179G>T | | | c.e6+686C>A | |  | | Q9NZJ7 | |
| ANKRD30A | | Missense | | SNP | | chr10:37490239G>A | | | c.3212G>A | | p.S1071N | | Q9BXX3 | |
| AGAP2 | | Missense | | SNP | | chr12:58125611C>T | | | c.926G>A | | p.R309H | | Q99490 | |
| TPP2 | | Missense | | SNP | | chr13:103328667C>T | | | c.3562C>T | | p.H1188Y | | P29144 | |
| IQGAP1 | | Intronic | | SNP | | chr15:90986819G>A | | | c.e9+109G>A | |  | | P46940 | |
| ATCAY | | Missense | | SNP | | chr19:3909578G>A | | | c.742G>A | | p.G248S | | Q86WG3 | |
| EVI5L | | Frameshift | | DEL | | chr19:7918216delC | | | c.1143delC | | p.R383fs | | Q96CN4 | |
| NF2 | | Frameshift | | DEL | | chr22:30032834_30032835delAC | | | c.209_210delAC | | p.T71fs | | P35240 | |
| NF2 | | Frameshift | | INS | | chr22:30032835_30032836insA | | | c.210_211insA | | p.T71fs | | P35240 | |
|  | | | | | | | | | | | | | | |
| **VSi-25-CasD7** | | | | | | | | | | | | | | |
| **Gene Symbol** | | **Variant Classification** | | **Variant Type** | | **Genome Change** | | | **cDNA Change** | | **Protein Change** | | **SwissProt acc_Id** | |
| WDR75 | | Intronic | | SNP | | chr2:190334990A>G | | | c.e17+19A>G | |  | | Q8IWA0 | |
| TSEN2 | | Missense | | SNP | | chr3:12571316C>T | | | c.1192C>T | | p.P398S | | Q8NCE0 | |
| IGSF10 | | Missense | | SNP | | chr3:151166779C>T | | | c.990G>A | | p.M330I | | Q6WRI0 | |
| AMTN | | Intronic | | SNP | | chr4:71384605C>A | | | c.e2+57C>A | |  | | Q6UX39 | |
| PAPSS1 | | Intronic | | SNP | | chr4:108565897T>C | | | c.e3-61A>G | |  | | O43252 | |
| TRGV1 | | RNA | | SNP | | chr7:38407419G>C | | | c.119C>G | |  | |  | |
| MAGI2 | | Intronic | | DEL | | chr7:77702356delG | | | c.e2-5909CA>A | |  | | Q86UL8 | |
| MAGI2 | | Intronic | | SNP | | chr7:77702357C>A | | | c.e2-5907G>T | |  | | Q86UL8 | |
| CLCN1 | | Splice_Site | | SNP | | chr7:143028744C>T | | | c.1165C>T | | p.H389Y | | P35523 | |
| DLC1 | | Intronic | | SNP | | chr8:13133754C>A | | | c.e14-29024G>T | |  | | Q96QB1 | |
| TMEM126B | | Missense | | SNP | | chr11:85345187A>G | | | c.261A>G | | p.I87M | | Q8IUX1 | |
| APPL2 | | Intronic | | SNP | | chr12:105623044G>T | | | c.e20+43C>A | |  | | Q06481 | |
| YY1 | | Nonsense | | SNP | | chr14:100706206C>T | | | c.625C>T | | p.Q209* | | P25490 | |
| SLC24A1 | | Missense | | SNP | | chr15:65938015G>T | | | c.2206G>T | | p.D736Y | | O60721 | |
| TICRR | | Intronic | | SNP | | chr15:90128887C>A | | | c.e4-52C>A | |  | | Q7Z2Z1 | |
| LGALS13 | | Intronic | | SNP | | chr19:40095199C>A | | | c.e2-43C>A | |  | | Q9UHV8 | |
| MARK4 | | Intronic | | SNP | | chr19:45767930C>G | | | c.e5-12C>G | |  | | Q96L34 | |
| CST2 | | Missense | | SNP | | chr20:23807073C>A | | | c.225G>T | | p.E75D | | P09228 | |
| NF2 | | Frameshift | | DEL | | chr22:30032834_30032835delAC | | | c.209_210delAC | | p.T71fs | | P35240 | |
| NF2 | | Frameshift | | INS | | chr22:30032835_30032836insA | | | c.210_211insA | | p.T71fs | | P35240 | |
| **VSi-267-CasD2** | | | | | | | | | | | | | | |
| **Gene Symbol** | | **Variant Classification** | | **Variant Type** | | **Genome Change** | | | **cDNA Change** | | **Protein Change** | | **SwissProt acc_Id** | |
| TTC34 | | Silent | | SNP | | chr1:2706547C>A | | | c.1149G>T | | p.L383L | | A8MYJ7 | |
| SIDT1 | | Intronic | | SNP | | chr3:113342242C>T | | | c.e22-32C>T | |  | | Q9NXL6 | |
| TRIM4 | | Intronic | | SNP | | chr7:99490380A>T | | | c.e1+11T>A | |  | | Q9C037 | |
| TRBV24OR9-2 | | RNA | | SNP | | chr9:33649340C>A | | | c.e2+29C>A | |  | |  | |
| DPH1 | | Intronic | | SNP | | chr17:1943961G>A | | | c.e9+62G>A | |  | | Q9BZG8 | |
| CCDC178 | | Intronic | | SNP | | chr18:30672889A>T | | | c.e3+15T>A | |  | | Q5BJE1 | |
| NF2 | | Frameshift | | DEL | | chr22:30032834_30032835delAC | | | c.209_210delAC | | p.T71fs | | P35240 | |
| **Table S5. *In vitro* iPSC differentiation assays** | | | | | | | | | | | | |  |  |
| ***NF2* Genotype** | | **+/-** | | **+/-** | | **+/-** | **-/-** | | **-/-** | | **-/-** | |  |  |
|  | |  | |  | | **NF2-FiPS-PM-B2** | **NF2-FiPS-MM-H6** | | **NF2-25iPS-MM-D7** | | **NF2-267iPS-MM-D2** | |  |  |
| ***In vitro* differentiation (EBs) Ectoderm** | | Tuj1 +  GFAP + | | Tuj1 +  GFAP + | | Tuj1 +  GFAP + | Tuj1 +  GFAP + | | EBs do not succeed | | EBs do not succeed | |  |  |
| ***In vitro* differentiation (EBs) Endoderm** | | AFP +  FOXA2 + | | AFP +  FOXA2 + | | AFP +  FOXA2 + | EBs do not succeed | | EBs do not succeed | | EBs do not succeed | |  |  |
| ***In vitro* differentiation (EBs) Mesoderm** | | ASMA +  GATA4 + | | ASMA +  GATA4 + | | ASMA +  GATA4 + | EBs do not succeed | | EBs do not succeed | | EBs do not succeed | |  |  |
| **Direct differentiation Ectoderm** | |  | |  | | **-** |  | | \| Tuj1 +  GFAP –  PAX6 + \|  \| \| --- \| --- \| | | Tuj1 +  GFAP –  PAX6 + | |  |  |
| **Direct differentiation Endoderm** | |  | |  | | **-** | AFP +  FOXA2 +  SOX17 + | | AFP +  FOXA2 +  SOX17 + | | AFP +  FOXA2 +  SOX17 + | |  |  |
| **Direct differentiation Mesoderm** | |  | |  | | **-** | ASMA +  GATA4 + | | ASMA +  GATA4 + | | ASMA +  GATA4 + | |  |  |
